# Pituitary Apoplexy: A Retrospective Study of 36 Cases From a Single Center

**DOI:** 10.7759/cureus.29769

**Published:** 2022-09-30

**Authors:** Sandra Arbunea-Ghenoiu, Gheorghe Vasile Ciubotaru, Anda Dumitrascu, Daniela Alexandrescu, Cristina Capatina, Catalina Poiana

**Affiliations:** 1 Endocrinology Department VI, “C.I. Parhon” National Institute of Endocrinology, Bucharest, ROU; 2 Neurosurgery Clinical Department III, Emergency Clinical Hospital “Dr. Bagdasar-Arseni”, Bucharest, ROU; 3 Radiology Department, “C.I. Parhon” National Institute of Endocrinology, Bucharest, ROU; 4 Ophthalmology Department, “C.I. Parhon” National Institute of Endocrinology, Bucharest, ROU; 5 Endocrinology Department, “Carol Davila” University of Medicine and Pharmacy, Bucharest, ROU

**Keywords:** treatment outcome, disease management, hypopituitarism, visual disturbance, pituitary apoplexy

## Abstract

Background and objective

Pituitary apoplexy (PA) is a possible life-threatening disorder due to spontaneous hemorrhage or impaired blood supply in the pituitary gland. It may present as an acute or subclinical form, and treatment options include either surgery or a conservative approach. The purpose of this study was to retrospectively analyze the clinical, imaging, and hormonal features, as well as the therapeutic outcomes, in a relatively short period of time in a series of consecutive patients with pituitary apoplexy (PA).

Results

Thirty-six patients were included, 50% presenting typical symptoms of PA. The presenting symptoms were headache (44.4%), visual abnormalities (44.4%), and digestive symptoms (22.2%). At diagnosis, hormonal deficiency was observed in 22 (61.1%) patients. Of the evaluated patients, 78.2% of the 23 operated cases and all unoperated cases presented tumor remnants. Vision improved in 81.8% of the operated and 100% of conservatively managed cases. Of all cases, 69.4% remained with long-term hypopituitarism.

Conclusion

Complex management of PA frequently leads to visual improvement but long-standing hypopituitarism.

## Introduction

First described by Bailey in 1898, pituitary apoplexy (PA) represents a rare condition characterized by abrupt hemorrhage and/or ischemia of the constituents of sella turcica, mainly concerning a pituitary adenoma [1-3]. The classical presentation consists of sudden onset of symptoms such as headache, vomiting, and visual disturbances, while the subclinical form refers to imaging signs of tumor hemorrhage without the clinical appearance previously mentioned [1,4]. Potential predisposing or precipitating factors for developing PA (such as arterial hypertension, previous surgery, or head trauma) may be associated [1,5]. Regarding the tumor type, PA is more likely to occur in nonfunctioning pituitary adenomas; most frequently, apoplexy involves a macroadenoma [1,5]. Because of the nonspecific signs and symptoms, the diagnosis of PA may be challenging and relies on the sequence of clinical presentation and imaging proof of the pituitary lesion [1,6,7]. Endocrine dysfunction may also be present; decreased levels of one or more anterior pituitary hormones are usually present before or occur at the onset of PA [1,6,7]. Cases of patients who develop diabetes insipidus are rare [1,8]. Sudden hemorrhage, accounting for the compression of optic nerves and chiasm, leads to visual field disturbances, and cranial nerve deficits are present in many cases [1,7]. The treatment management of PA includes two strategies: a surgical approach and a conservative approach. The main challenge is to establish if the patient benefits from the surgical intervention [6,8]. The impact of the choice of management on the outcome of these patients (including ocular disturbances, pituitary function, and pituitary tumor) is of paramount importance, and a multidisciplinary team must carefully evaluate each case [1,9,10].

The objective of our study was to analyze predisposing or precipitating factors, clinical status, imaging and hormonal features, and therapeutic management and its outcome for 36 cases with PA evaluated in the course of one year in a single tertiary care endocrinological center. The rationale of our study came from the lack of an existing guideline for the treatment of PA based on previous studies, so adding our experience to the literature may help elaborate specific recommendations regarding the management of these cases.

## Materials and methods

We performed a retrospective study that included 36 patients (sample size determination) diagnosed with PA and examined for one year (from January 1, 2019, to December 31, 2019) in the Department of Pituitary and Neuroendocrine Pathology at the “C.I. Parhon” National Institute of Endocrinology, Bucharest, Romania. The inclusion criteria regarding PA diagnosis divided our group of patients into two categories: the ones with classical clinical presentation, i.e., patients exhibiting signs of acute typical manifestations (e.g., headache and visual disturbances) accompanied by imagistic proof of hemorrhage or tumor necrosis, and the ones with subclinical presentation defined as brain imaging evidence of pituitary adenoma hemorrhage in asymptomatic/oligosymptomatic patients. The exclusion criteria included patients with a lack of conclusive investigations. Tumor hemorrhage was histopathologically confirmed in all operated cases. In all cases, a full pituitary function evaluation (measurement of serum levels of anterior pituitary hormones, free thyroxine (fT4), testosterone/estradiol, cortisol, corticotropin stimulation test when needed, insulin-like growth factor 1 (IGF-1), growth hormone (GH) suppression test when needed, urine osmolality, and water deprivation test when needed), formal ophthalmological evaluation, and pituitary imaging (magnetic resonance imaging (MRI) or computed tomography (CT)) were performed at diagnosis and during follow-up. Close endocrinological and ophthalmological follow-up was performed. Hypopituitarism was established according to the current criteria of diagnosis [11]. Pituitary Apoplexy Score (PAS), which represents a clinical staging system including the level of consciousness, visual acuity, and field defects and ocular palsies, was retrospectively calculated [1]. Data collected from patient files included the gender of the patient, age at diagnosis, medical history, actual or past treatments, prior signs or symptoms before the apoplectic event, clinical status at diagnosis, visual status, imaging features described on CT or MRI scans, standard blood tests and full endocrine evaluation, and outcomes after therapeutic management. However, medical history (including past treatments) was not complete in all of the patients. Data analysis was done using Microsoft Excel (Microsoft Corp., Redmond, WA, USA).

## Results

Clinical presentation and predisposing events

The study included 19 males and 17 females. The mean age at diagnosis was 49.2 years (range: 14-72 years). Nine (25%) patients were previously known to have a pituitary adenoma (three of these patients presented a classical episode of PA, and six of these patients had an imagistic diagnosis unaccompanied by typical clinical manifestations). These nine cases include five nonfunctioning pituitary adenomas, three prolactinomas, and one adrenocorticotropic hormone (ACTH)-secreting pituitary adenoma. Two of the nine cases underwent surgery before the apoplectic event, and all patients with prolactinoma were under treatment with dopamine agonists (DAs) at the time of PA. Regarding the type of pituitary adenoma in the patients who were not previously known to have a pituitary tumor (27), one newly discovered secreting pituitary tumor was found after the PA diagnosis was established (GH-secreting pituitary adenoma).* *Half of the patients (18) presented a classical PA episode (eight of these patients have at least one precipitating factor before the apoplectic event), while the other half had pituitary adenoma hemorrhage described on sellar imaging accompanied by minimal, atypical symptoms (seven of them presented at least one precipitating factor prior to the apoplexy diagnosis). Overall, 41.6% of our patients had at least one possible predisposing factor (Table 1).

**Table 1:** Precipitating factors for PA in our cohort of patients PA: pituitary apoplexy

Precipitating factor	Total (number of patients (% of the total))	Classical PA (number of patients (% of the total))	Subclinical PA (number of patients (% of the total))
Arterial hypertension	8 (22.2%)	6 (16.6%)	2 (5.5%)
Angiographic or surgical procedures	2 (5.5%)	1 (2.7%)	1 (2.7%)
Head trauma	2 (5.5%)	1 (2.7%)	1 (2.7%)
Dopamine agonist treatment	3 (8.3%)	0 (0%)	3 (8.3%)
Anticoagulation therapy	1 (2.7%)	1 (2.7%)	0 (0%)

Eight patients retrospectively recognized various signs or symptoms possibly suggestive of pituitary pathology present before the apoplectic event (Table 2).

**Table 2:** Signs and symptoms of pituitary dysfunction present before PA PA: pituitary apoplexy

Prior signs and symptoms	Total (number of patients (% of the total))	Classical PA (number of patients (% of the total))	Subclinical PA (number of patients (% of the total))
Menstrual or sexual disturbances	5 (29.4% of women)	2 (5.5%)	3 (8.3%)
Past episodes of headache	4 (11.1%)	1 (2.7%)	3 (8.3%)
Fatigability	1 (2.7%)	1 (2.7%)	0 (0%)
Galactorrhea	1 (2.7%)	0 (0%)	1 (2.7%)

The most common symptoms of apoplexy in all of our patients (including the subclinical cases with mild symptoms) were headache (44.4%), visual abnormalities (44.4%), and digestive manifestations (22.2%). Regarding the most common manifestations of apoplexy in the 18 patients presenting with a sudden and severe onset of symptoms, headache and ocular disorders were the most common (72.2% each), followed by digestive phenomena (38.8%). Oculomotor palsies were found in nine patients with classical PA. Mild similar symptoms were described in the subclinical group (Table 3).

**Table 3:** Clinical presentation of PA PA: pituitary apoplexy

Clinical picture at diagnosis	Total (number of patients (% of the total))	Classical PA (number of patients (% of the total))	Subclinical PA (number of patients (% of the total))
Headache	16 (44.4%)	13 (36.1%)	3 (8.3%)
Visual disturbances	16 (44.4%)	13 (36.1%)	3 (8.3%)
Oculomotor palsies	10 (27.7%)	9 (25%)	1 (2.7%)
Decreased visual acuity	7 (19.4%)	4 (11.1%)	3 (8.3%)
Visual field defects	8 (22.2%)	5 (13.8%)	3 (8.3%)
Vomiting and nausea	8 (22.2%)	7 (19.4%)	1 (2.7%)
Fatigability	2 (5.5%)	1 (2.7%)	1 (2.7%)
Pilosity or skin changes	2 (5.5%)	0 (0%)	2 (5.5%)
Sexual dysfunction	1 (2.7%)	0 (0%)	1 (2.7%)
Gynecomastia	2 (5.5%)	0 (0%)	2 (5.5%)
Arterial hypertension	1 (2.7%)	1 (2.7%)	0 (0%)
Others (weight loss, sweating, loss of appetite, balance disorder, and coma)	6 (16.6%)	4 (11.1%)	2 (5.5%)

Pituitary Apoplexy Score (PAS)

PAS was determined retrospectively for 30 patients (incomplete required available data for calculation in six cases): 17 patients who underwent surgery (73.9%) and all 13 patients who were managed conservatively. An important number of patients (11) were attributed zero points, and the majority presented with PAS ≤ 4 points (82.3% of the operated patients and 92.3% of the patients who had a conservative management approach) (Table 4). PAS was not taken into consideration in our patients when a therapeutic choice was made.

**Table 4:** PAS score in our patients PAS: Pituitary Apoplexy Score

PAS	Neurosurgery (number of patients)	Conservative management (number of patients)
0	5	6
1	1	1
2	3	2
3	2	0
4	3	3
5	1	0
6	2	1

Sellar imaging: radiological features at diagnosis

As previously mentioned, only nine (25%) cases were previously known to have a pituitary adenoma: five nonfunctioning pituitary adenoma, three prolactinomas, and one ACTH-secreting pituitary adenoma. Eighteen patients were evaluated using MRI alone, 10 patients underwent CT scans alone, and both investigations were performed for eight patients. Pituitary macroadenomas were diagnosed in 25 (69.4%) cases, four patients presented pituitary adenoma under 10 mm maximum diameter, and no available data regarding tumor dimension was mentioned for seven patients.

Hormonal assessment

When PA diagnosis was established, 22 patients were diagnosed with hypopituitarism (five of them were already known to have one or combined pituitary hormone deficiency; in the others, no prior evaluation was available due to the lack of significant suggestive symptoms). Corticotropic deficiency was noted in seven cases and was the most common deficit in patients with classical PA (4/7 patients, 57.1%), while gonadotropic deficiency had the greatest prevalence in subclinical cases (6/9 patients, 66.6%).

Management and outcomes

Twenty-three (63.8%) patients underwent neurosurgical intervention; 11 of them have typical clinical presentations. Twelve patients with a subclinical form of PA underwent delayed surgery, mostly within one month from diagnosis. Neurosurgical decompression was performed (either for tumor debulking or due to the lack of satisfactory response to initial conservative management). The decision was made by a multidisciplinary team (endocrinologist, ophthalmologist, radiologist, and neurosurgeon).

Tumor Outcome

**Figure 1:**
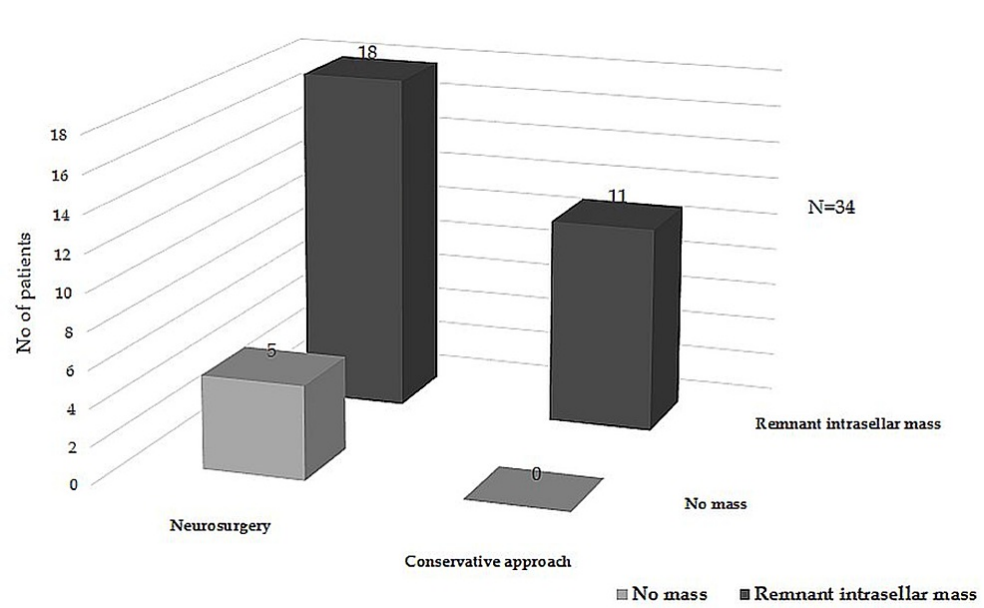
Evolution of pituitary mass during follow-up

After the apoplectic event, imaging follow-up results showed that the majority of the evaluated patients by CT/MRI presented remnant intrasellar mass (85.3%), irrespective of the management type (78.2% of those operated and 100% of those conservatively managed) (Figure 1). Two patients are yet to be evaluated (both were treated conservatively).

Visual Outcome

**Figure 2:**
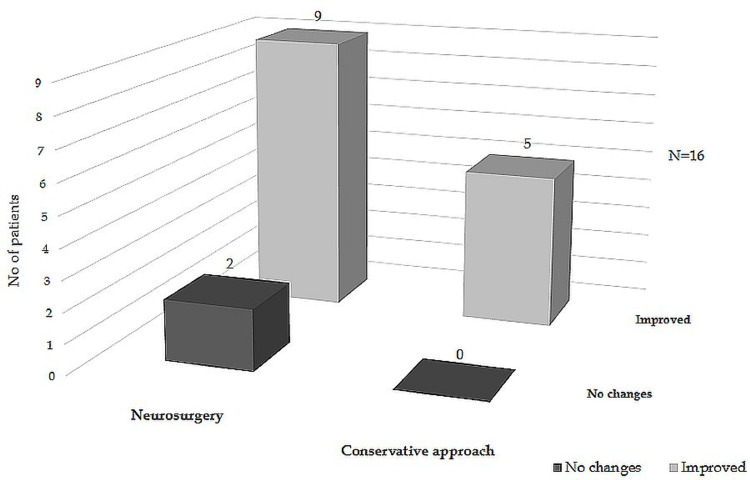
Evolution of visual function during follow-up

Out of 16 cases with visual disturbances, 11 patients were operated on, and five were managed conservatively. Nine (81.8%) of the operated patients and all cases with conservative treatment had improvement in vision (Figure 2).

Hormonal Outcome

**Figure 3:**
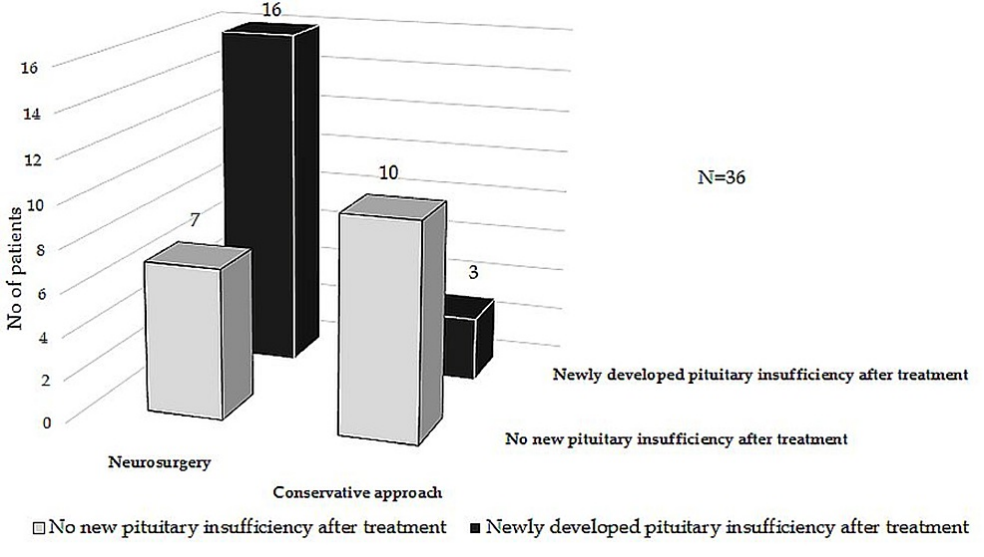
Evolution of pituitary function during follow-up

Newly developed pituitary insufficiency was more important in patients undergoing surgery versus patients treated conservatively (Figure 3). A total of 25 (69.4%) patients remained with long-term hormone replacement therapy. Four (11%) cases developed postoperative diabetes insipidus (three transient and one permanent). Our cohort included one patient previously diagnosed with Cushing’s disease who presented resolution of this syndrome after PA.

## Discussion

We have retrospectively analyzed the clinical course and therapeutic outcome for 36 patients with PA referred to our center during 2019. The mean age at diagnosis in our patients is similar to previous studies [1]. We noted a slightly male preponderance in our cohort, also observed in other reports [2,5,8,9,12-15]. Our results show that there is an equal distribution among patients regarding the clinical type of the syndrome (acute presentation versus subclinical form), while other studies establish a higher frequency of the subclinical type of event [1,16]. The percentage of predisposing or precipitating factors found in our patients is similar to other studies [2,3,8,13]. Arterial hypertension was described as a possible precipitating factor [17,18], but there is no certain evidence so far for the association between chronic high blood pressure and the appearance of an apoplectic event [3,5]. Investigations contributing to blood pressure fluctuations (angiography and surgery) were less common in our patients, but some reports suggest that variation in blood pressure may precipitate PA [3,16]. We identified a history of dopamine agonist treatment in a few cases, all presenting with subclinical pituitary hemorrhage. Some [12], but not all [5], authors consider this treatment a potential risk factor for PA.

Inconsistent symptoms of pituitary disorder were retrospectively found in a minority of our patients (menstrual/sexual disturbances, past episodes of severe headache, fatigability, and galactorrhea) a few months before the diagnosis of PA, mostly of the subclinical type. Similar clinical features suggestive of endocrine dysfunction were also described as being rare in other cohorts [18]. Other studies broadly reflect comparable aspects of the clinical presentation at diagnosis [4,7-9,13,16,19]. Headache and visual impairment are the most commonly reported symptoms in the affected patients, in many cases accompanied by nausea or vomiting. In our study, the mentioned symptoms were mostly found in patients presenting with the acute form of PA. Liu et al. detected hormone dysfunction as the second most common symptom (after visual impairment) in the subclinical group of patients [16]. Oculomotor palsies are frequent in our cohort, and a similar percentage was found in other studies [1].

Although the Pituitary Apoplexy Score (PAS) suggested by the UK Pituitary Apoplexy Guidelines Development Group was not calculated and taken into consideration in our patients before the therapeutic decision, it could represent a solution for better division of cases when defining a major neuro-ophthalmic insult and therefore making the best choice between management options. In the future, the use of this type of tool may improve the follow-up and perhaps help elaborate guidelines for the optimal management of PA [1,6,9].

In our series, both CT and MRI were used to diagnose pituitary lesions. For some of our patients, an MRI scan was used even after a CT examination was made, which supports what other authors claim about the fact that CT is not sensitive enough for PA diagnosis and the accurate assessment of the contents of the pituitary sella [2,13,18]. Most patients presented remnant intrasellar mass after therapy. Long-term imaging follow-up is needed in our patients to compare tumor outcomes between patients receiving surgery and those conservatively treated after apoplexy. Ayuk et al. mentioned that tumor regrowth is not frequent,and it is comparable in both types of management [2], while Sibal et al. stated that the incidence is lower in operated cases [13]. The design of our study did not allow long-term follow-up of the tumor remnant.

Patients affected by PA were diagnosed with a nonfunctioning pituitary adenoma in most of our cases as in other studies [1]; perhaps an explanation of this phenomenon consists of the large area of necrosis in the adenoma, which makes it impossible to assess whether the tumor was releasing hormones or not before the apoplectic event.

In the classical symptomatic form of PA, corticotropic deficiency is considered the most common acute endocrine dysfunction [1,2,4,5,8,12,13,18,20]. We discovered that gonadotropic deficiency is the main deterioration in the pituitary function in subclinical cases, although in one study, in this type of apoplexy, the pituitary function was not affected [20]. Data collected after treatment shows in our study that half of the patients developed a new pituitary insufficiency and most of those underwent surgery, as opposed to the findings of Randeva et al., who reported restoration of pituitary function in more than half of the patients after surgery [18]. We found normalization of pituitary function in only two patients after surgery. Regarding the one case previously diagnosed with Cushing’s disease, two similar cases of apparent biochemical cure after PA were presented in one study, and both had a recurrence of the disease in the next months [13]. Long-term follow-up of our patients is essential to observe the possible further improvement or deterioration of the endocrine function. Visual function in most of our patients was improved after treatment in both conservative and surgically treated categories, with better results in the conservative management group. This data is similar to other studies [2,9,13], and the selection bias is probably at least in part responsible (surgery is usually considered when a severe visual disturbance is present at diagnosis) [1].

The main limits of our study are represented by the retrospective nature and relatively small sample of patients. Also, the retrospective calculation of the PAS might not prove to be accurate in all patients. The design of our study did not allow long-term follow-up of the tumor remnant.

## Conclusions

Pituitary apoplexy is a rare and challenging endocrine condition that can be difficult to diagnose and treat. We consider achieving the objective of our study, and our verdict is in large part similar to preexisting literature. The visual recovery in these patients is notable, but the outcome of pituitary function is less encouraging. As no consensus guidelines of optimal management are available, our study offers a global image and raises awareness about the management of this disorder in our country and underlines the need for multidisciplinary evaluation of these patients and future practice guidelines to optimize specialized care for these rare patients.
